# Cerebral Perfusion Measurements in Elderly with Hypertension Using Arterial Spin Labeling

**DOI:** 10.1371/journal.pone.0133717

**Published:** 2015-08-04

**Authors:** H. J. M. M. Mutsaerts, J. W. van Dalen, D. F. R. Heijtel, P. F. C. Groot, C. B. L. M. Majoie, E. T. Petersen, E. Richard, A. J. Nederveen

**Affiliations:** 1 Department of Radiology, Academic Medical Center, Amsterdam, The Netherlands; 2 Department of Neurology, Academic Medical Center, Amsterdam, The Netherlands; 3 Danish Research Centre for Magnetic Resonance, Centre for Functional and Diagnostic Imaging and Research, Copenhagen University Hospital Hvidovre, Hvidovre, Denmark; 4 Department of Neurology, Radboud University Medical Centre, Nijmegen, The Netherlands; Henry Ford Health System, UNITED STATES

## Abstract

**Purpose:**

The current study assesses the feasibility and value of crushed cerebral blood flow (CBF_crushed_) and arterial transit time (ATT) estimations for large clinical imaging studies in elderly with hypertension.

**Material and Methods:**

Two pseudo-continuous arterial spin labeling (ASL) scans with (CBF_crushed_) and without flow crushers (CBF_non-crushed_) were performed in 186 elderly with hypertension, from which CBF and ATT maps were calculated. Standard flow territory maps were subdivided into proximal, intermediate and distal flow territories, based on the measured ATT. The coefficient of variation (CV) and physiological correlations with age and gender were compared between the three perfusion parameters.

**Results:**

There was no difference in CV between CBF_crushed_ and CBF_non-crushed_ (15–24%, *p*>0.4) but the CV of ATT (4–9%) was much smaller. The total gray matter correlations with age and gender were most significant with ATT (*p* = .016 and *p*<.001 respectively), in between for CBF_crushed_ (*p* = .206 and *p* = .019) and least significant for CBF_non-crushed_ (*p* = .236 and *p* = .100).

**Conclusion:**

These data show the feasibility and added value of combined measurements of both crushed CBF and ATT for group analyses in elderly with hypertension. The obtained flow territories provide knowledge on vascular anatomy of elderly with hypertension and can be used in future studies to investigate regional vascular effects.

## Introduction

Perfusion as measured with arterial spin labeling (ASL) is a promising *in vivo* hemodynamic parameter to investigate the interplay between normal aging and neurodegenerative and cerebrovascular pathology [1,2]. Parallel to the optimization of conventional ASL-based cerebral blood flow (CBF) measurements for clinical applications, advanced ASL methods have been developed that enable the acquisition of multiple perfusion parameters simultaneously [3–5].

**Fig 1:**
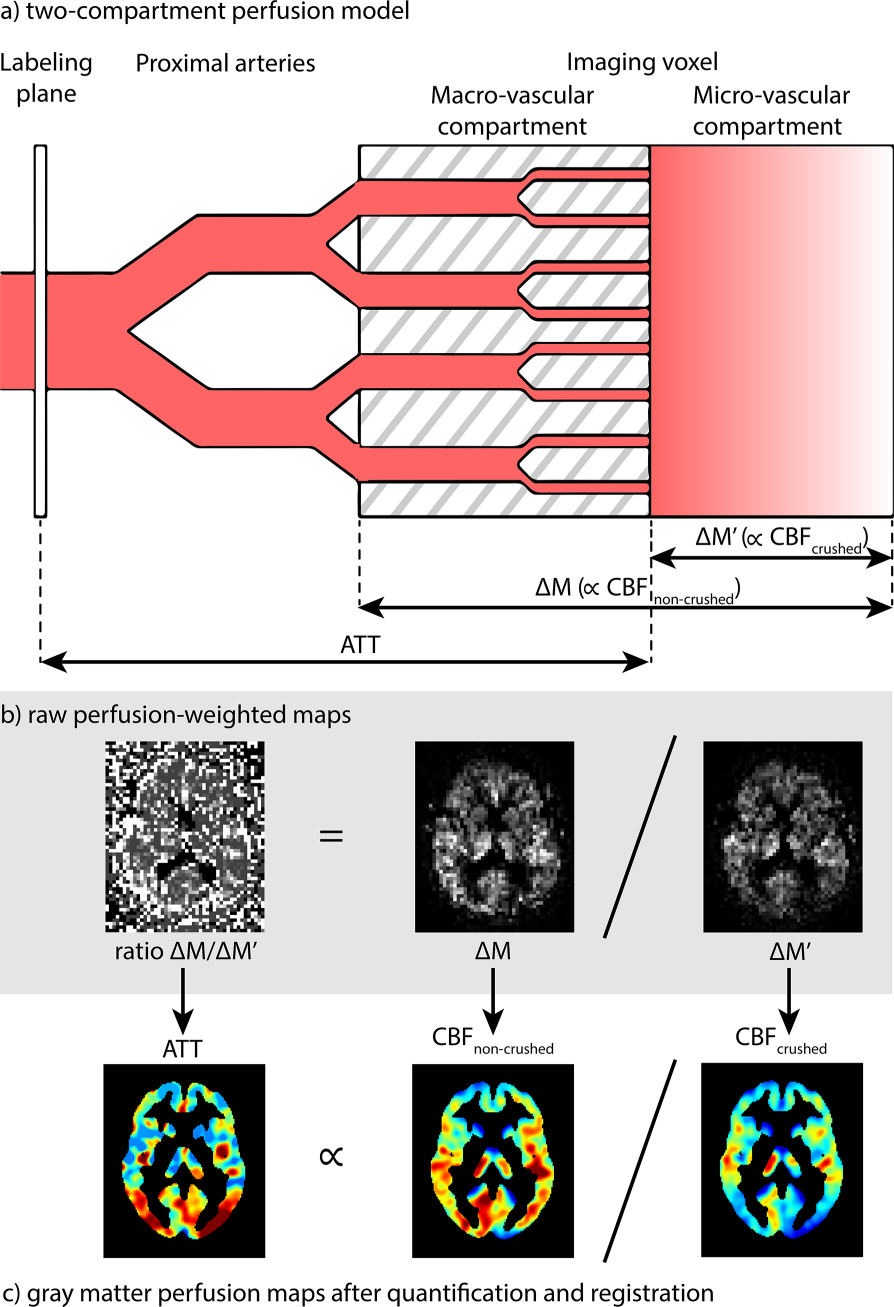
a) Schematic overview of the two-compartment perfusion model explains the FEAST technique, adapted from Wang *et al*. [6] b) shows raw perfusion-weighted maps and c) perfusion maps after post-processing for a representative subject. Note that the signal intensity is lower after crushing (ΔM') than before (ΔM) and that crushed CBF (CBF_crushed_) is weighted toward the micro-vascular CBF whereas non-crushed CBF (CBF_non-crushed_) is weighted toward both micro- and macro-vascular CBF. ATT = arterial transit time, ∝ = proportional to.

One example is the acquisition of both CBF and the micro-vascular arterial transit time (ATT) by the flow-encoding arterial spin tagging (FEAST) method. FEAST is based upon the subdivision of an imaging voxel into macro- and micro-vascular compartments based on differences in blood flow velocity [6]. By performing ASL with and without the application of a vascular crusher, FEAST separately acquires CBF of the micro-vascular compartment (CBF_crushed_) and CBF of the macro- and micro-vascular compartments together (CBF_non-crushed_) (Fig 1). The ratio of the perfusion signal of CBF_non-crushed_ over CBF_crushed_ is then proportional to the ATT, which is defined as the time it takes for the labeled blood to travel from the labeling plane to the micro-vascular compartment of the imaging voxel.

Several advantages exist for the application of a vascular crusher and the estimation of ATT using FEAST. CBF_crushed_ may be preferable to CBF_non-crushed_, since micro-vascular CBF changes are generally assumed to reflect (patho-)physiological changes in neuronal activity and energy demand whereas macro-vascular CBF is believed to be dominated by cardiovascular fluctuation [7]. ATT has been shown to be able to provide additional diagnostic value to CBF measurements, especially in cerebrovascular pathology [8,9]. Compared to the measurement of ATT with multiple time point approaches (multi-TI), advantages of FEAST include whole-brain coverage, relatively high SNR [10], relatively low motion sensitivity and relatively low computational demand [6]. However, these possible advantages may come at the cost of a decrease of reliability. Considering the often limited available scanning time, vascular crushing reduces the available SNR for the CBF measurement [6]. Therefore, it has been recently agreed upon that crushing is currently not recommended in the individual subject [11]. However, for group analyses, the feasibility and utility of crushed CBF is still under debate. Moreover, the ATT range that can be reliably measured by FEAST is restricted and dependent upon an a-priori ATT estimate.

The primary purpose of this study was to assess the feasibility and value of the combined measurement of CBF_crushed_ and ATT for large clinical imaging studies in elderly with hypertension. To investigate the spatial distribution of these parameters, we used the estimated ATTs to divide standard flow territory maps into proximal, intermediate and distal flow territories. We study the reliability of these perfusion parameters in terms of their population variation and physiological correlations with age and gender.

## Materials and Methods

### Subjects

195 community-dwelling elderly (46% male, mean age 77 years, range 72–80 years) with hypertension (systolic blood pressure higher than 140 mmHg) participating in the Pre-DIVA study were eligible for inclusion [12]. Exclusion criteria were dementia and disorders or circumstances expected to interfere with successful follow-up. Nine subjects were excluded from analysis because of severe labeling or motion artifacts.

### Ethics statement

All patients provided written informed consent and the study was approved by the institutional review board of the Academic Medical Center, Amsterdam.

### Imaging protocol

All imaging was performed on a 3T system (Intera, Philips Healthcare, Best, The Netherlands) equipped with an 8-channel head coil. Foam padding was used to restrict head motion. A slightly adapted version of the original FEAST acquisition, which enables the simultaneous acquisition and quantification of CBF_crushed_, CBF_non-crushed_ and ATT within clinical scanning time, was added to a routine clinical dementia protocol. Two ASL scans were performed with (CBF_crushed_) and without (CBF_non-crushed_) flow-crushing gradients in three directions (velocity encoding 50 mm/s). Identical imaging parameters of the two consecutive gradient-echo single-shot EPI pseudo-continuous ASL (pCASL) sequences were: matrix = 64x64, FOV = 240 x 240 mm, 17 axial slices, slice thickness 7 mm, no gap, echo time/repetition time = 17/4000 ms, flip angle = 90 degrees, SENSE = 2.5, initial post-label delay (PLD) = 1525 ms; slice readout time = 34.9 ms; resulting PLD range for 17 slices = 1525–2080 ms, labeling duration = 1650 ms and two background suppression pulses at 1710 and 2860 ms after a pre-labeling saturation pulse. 20 label-control pairs were acquired ‒ resulting in a duration of 2:40 minutes for each scan. The labeling plane was positioned parallel and 8.3 cm inferior to the center of the imaging volume [13]. An isotropic 1 mm^3^ 3D T1-weighted scan was included in the imaging protocol for segmentation and registration purposes.

### Quantification

The raw EPI control and label images were 3D motion corrected using SPM8 (Statistical Parametric Mapping, Wellcome Trust Centre for Neuroimaging, London, UK). After pair-wise subtraction, these raw maps were converted to CBF with a single compartment model, assuming that the label decays with the T1 of blood [11,14]:

$$CBF[mL/100g/\text{min}]=\frac{\Delta Me^{TE/T_{2a}^{*}}}{\rho M_{0a}2\alpha \alpha_{inv}T_{1a}(e^{-\delta /T_{1a}}-e^{(-PLD-\tau )/T_{1a}})}$$ (1)

where ρ is the density of brain tissue (1.05 g/mL) [15], ΔM is the difference between control and label intensities, TE is the echo time (17 ms), T_2_*_a_ is the transverse relaxation time of arterial blood (50 ms) [16], M0_a_ is the equilibrium magnetization of arterial blood, for which an average scanner value was used [17], calculated according to previously described methods [18], α is the labeling efficiency (0.85) [13], α_inv_ is the correction for label loss due to background suppression pulses (0.83) [19], T_1a_ is the T_1_ relaxation time of arterial blood (1650 ms) [20], PLD = 1525 ms + 34.9 ms/slice, τ is the labeling duration (1650 ms) and δ is the measured ATT (averaged per subject for each flow territory, as described below) for CBF_crushed_, which is replaced by the PLD (1525 ms + 34.9 ms/slice) for CBF_non-crushed_.

ATT was calculated based on the following two FEAST equations [6]:

$$\Delta M\;=A\;(e^{-PLD/{T1}_{a}}-\;e^{\left(-PLD-\tau \right)/{T1}_{a}})$$ (2)

$$\Delta M\prime =A\;(e^{-\delta /{T1}_{a}}-\;e^{\left(-PLD-\tau \right)/{T1}_{a}})$$ (3)

where A is a constant and ΔM and ΔM' represent the scans acquired without and with vascular crushing, respectively (Fig 1). PLD slice timing differences were accounted for (1525 ms + 34.9 ms/slice).

### Registration

The 3D T1-weighted anatomical scans were segmented using SPM8 into gray matter tissue probability maps. After pair-wise subtraction, the CBF_crushed_ maps were rigid-body registered on the CBF_non-crushed_ maps. The CBF_non-crushed_ maps were rigid-body registered on the gray matter probability maps, and the same transformation was applied to the CBF_crushed_ maps. The tissue probability maps were spatially normalized using the Diffeomorphic Anatomical Registration analysis using Exponentiated Lie algebra (DARTEL) algorithm and the resulting normalization fields were applied to the CBF and ATT maps [21].

### Flow territories

The total cerebral gray matter was defined as tissue probabilities >70%. Standard flow territory templates (left and right combined) were used to investigate vascular territories supplied by the bilateral anterior, middle and posterior cerebral arteries (referred to as ACA, MCA and PCA respectively) [22]. Within each flow territory the ATTs were ranked in tertiles, resulting in three proximal, three intermediate and three distal flow territories.

### Statistics

Prior to all analyses, the distributions of investigated values were tested for normality using the Shapiro-Wilk test. Because most distributions of perfusion parameters deviated from normal, distributions were summarized by the median and mean absolute deviation (instead of mean and standard deviation). To compare variation between the perfusion parameters, the coefficient of variation (CV) was used, calculated as mean absolute deviation divided by the median. To test whether respectively the median or CV differed between CBF_crushed_ and CBF_non-crushed_, the sign test and Brown-Forsythe test were used (comparable to Student's t-test and Levene's test but more robust in distributions that deviate from normality). To provide insight in the distribution of CBF and ATT values in the ACA, MCA and PCA, median group-level histograms were generated from the histograms of individual maps (100 bins, smoothed with 2 bins full-width-half-maximum). To investigate physiological correlations, robust linear regression analyses were performed to model cross-sectional correlations between the predictors age or gender and the dependent variables CBF_non-crushed_, CBF_crushed_ or ATT, adjusted for total brain volume (defined as the combined volume of gray and white matter segmentations).

## Results

### Flow territories

**Fig 2:**
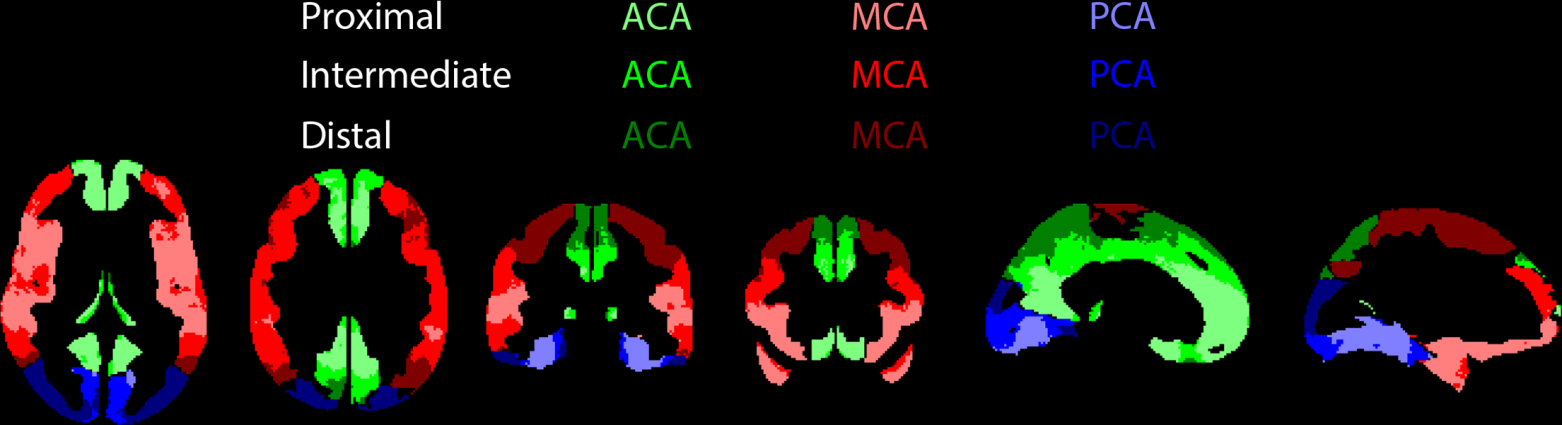
Flow territories. ACA (green), MCA (red) and PCA (blue) refer to the standard flow territories perfused by the bilateral anterior, middle and posterior cerebral arteries respectively, whereas the shadings represent their subdivision into proximal, intermediate and distal flow territories, based on arterial transit times.

The ATT-based flow territories showed an almost entirely continuous sequence from anterior-inferior to posterior-superior (ACA), inferior to superior (MCA) and anterior-inferior to posterior-superior (PCA) (Fig 2).

### Comparison perfusion parameters

**Fig 3:**
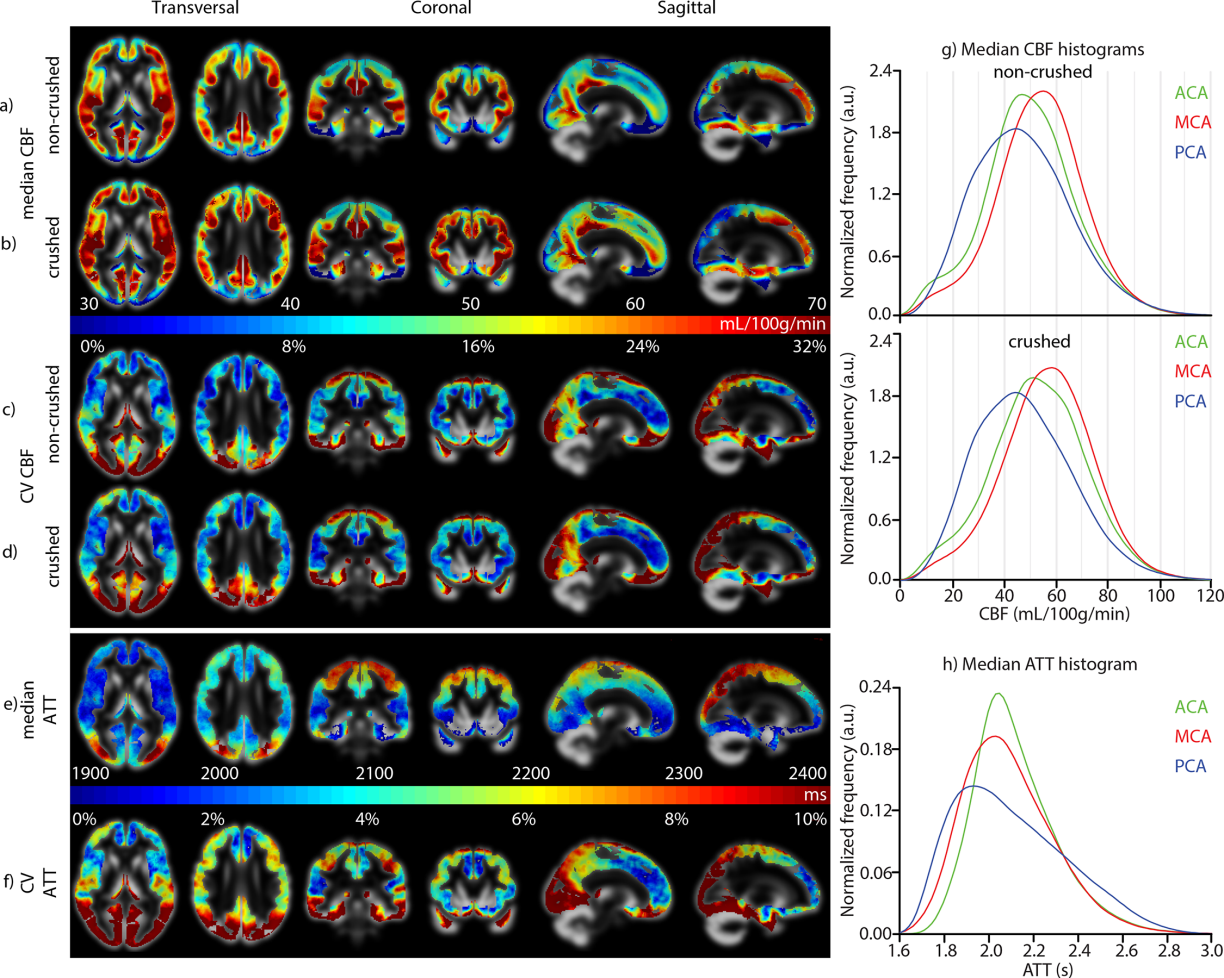
a-b) median and c-d) coefficient of variation (CV) maps of non-crushed cerebral blood flow (CBF) and crushed CBF, e) median and f) CV maps of arterial transit time (ATT). g,h) median histograms of (non-) crushed CBF and ATT maps for the three vascular territories. ACA, MCA and PCA refer to the vascular territories perfused by the anterior, middle and posterior cerebral arteries respectively, corresponding to Fig 2.

Fig 3 shows the median and CV maps of the perfusion parameters. The perfusion patterns in the median CBF_crushed_ and CBF_non-crushed_ maps had a similar appearance. Whereas the median CBF_crushed_ differed in nearly all flow territories from the median CBF_non-crushed_, there was no CV difference between CBF_crushed_ and CBF_non-crushed_ (*p*>0.4 all flow territories), both ranging from 15% to 20% (Table 1). The CV of ATT was much smaller than the CV of CBF_crushed_ or CBF_non-crushed_, ranging from 4% to 9%. For all perfusion parameters, CV increased from proximal to distal flow territories (Table 1). Whereas the CBF histograms approximated a normal distribution, the ATT histograms appeared skewed, especially the PCA (Fig 3H).

**Table 1:** Distributions of perfusion parameters (n = 186).

	CBF_non-crushed_ (CV) [mL/100g/min]	CBF_crushed_ (CV) [mL/100g/min]	Difference median ∙∙ CV	ATT (CV)[ms]
ACA proximal	48.4 ± 8.5 (17%)	52.8 ± 9.1 (17%)	Y ∙∙ N	1990 ± 90 (4%)
intermediate	53.8 ± 9.0 (17%)	58.0 ± 9.1 (16%)	Y ∙∙ N	2070 ± 100 (5%)
distal	46.8 ± 9.0 (19%)	49.6 ± 9.4 (19%)	Y ∙∙ N	2210 ± 130 (6%)
MCA proximal	53.5 ± 8.8 (17%)	57.8 ± 8.7 (15%)	Y ∙∙ N	1930 ± 80 (4%)
intermediate	54.8 ± 9.3 (17%)	58.5 ± 9.4 (16%)	Y ∙∙ N	2040 ± 110 (5%)
distal	53.9 ± 9.7 (18%)	55.7 ±10.0 (18%)	Y ∙∙ N	2210 ± 140 (6%)
PCA proximal	45.2 ± 8.5 (19%)	46.8 ± 8.9 (19%)	Y ∙∙ N	1930 ± 140 (7%)
intermediate	45.9 ±10.1 (22%)	46.7 ±10.4 (22%)	Y ∙∙ N	2030 ± 170 (9%)
distal	46.8 ±10.9 (23%)	46.3 ±10.9 (24%)	N ∙∙ N	2290 ± 200 (9%)
Total gray matter	51.4 ± 8.6 (17%)	54.6 ± 8.9 (16%)	Y ∙∙ N	2080 ± 110 (5%)

Shown are the median ± mean absolute deviation from median (with the coefficient of variation (CV) between parentheses) of cerebral blood flow (CBF) and arterial transit time (ATT). ACA, MCA and PCA refer to the flow territories perfused by the anterior, middle and posterior cerebral arteries respectively, corresponding to Fig 2. Difference (4^th^ column) shows whether the median or CV differed (Y) or not (N) (*p*<0.01) between CBF_crushed_ and CBF_non-crushed_.

### Physiological correlations

In all vascular territories, CBF_crushed_ and CBF_non-crushed_ decreased and the ATT increased with age, although the changes with age were significant in some flow territories only (Table 2). In almost all flow territories, men had lower CBF and longer ATTs compared to women. Generally, correlations were strongest with ATT and slightly stronger with CBF_crushed_ than with CBF_non-crushed_. The regression coefficients roughly suggested that both aging 10 years and being male decreases total gray matter CBF with 8% and increases total gray matter ATT with 5%. For all perfusion parameters, correlation coefficients and *p*-values increased and decreased respectively from proximal to distal flow territories.

**Table 2:** Regression coefficients for age and gender (n = 186).

	CBF_non-crushed_	CBF_crushed_	ATT
**Age**	[mL/100g/min/decade]	[mL/100g/min/decade]	[ms/decade]
ACA proximal	- 3.08 ∙∙ *p* = .334	- 4.11 ∙∙ *p* = .226	70 ∙∙ *p* = .033
intermediate	- 3.43 ∙∙ *p* = .316	- 2.92 ∙∙ *p* = .389	80 ∙∙ *p* = .015
distal	- 5.21 ∙∙ *p* = .138	- 5.89 ∙∙ *p* = .112	110 ∙∙ *p* = .013
MCA proximal	- 2.93 ∙∙ *p* = .392	- 2.36 ∙∙ *p* = .484	50 ∙∙ *p* = .082
intermediate	- 2.42 ∙∙ *p* = .501	- 2.63 ∙∙ *p* = .466	70 ∙∙ *p* = .088
distal	- 4.65 ∙∙ *p* = .219	- 5.31 ∙∙ *p* = .172	110 ∙∙ *p* = .036
PCA proximal	- 6.32 ∙∙ *p* = .052	- 6.74 ∙∙ *p* = .042	130 ∙∙ *p* = .008†
intermediate	-10.07 ∙∙ *p* = .008†	-10.68 ∙∙ *p* = .006†	180 ∙∙ *p* = .006†
distal	- 9.58 ∙∙ *p* = .025	- 8.81 ∙∙ *p* = .038	170 ∙∙ *p* = .020
Total gray matter	- 3.96 ∙∙ *p* = .236	- 4.31 ∙∙ *p* = .206	100 ∙∙ *p* = .016
**Gender**	[mL/100g/min M>F]	[mL/100g/min M>F]	[ms M>F]
ACA proximal	- 3.00 ∙∙ *p* = .102	- 4.93 ∙∙ *p* = .011	50 ∙∙ *p* = .003†
intermediate	- 4.67 ∙∙ *p* = .017	- 5.56 ∙∙ *p* = .004†	90 ∙∙ *p* < .001†
distal	- 4.20 ∙∙ *p* = .036	- 5.12 ∙∙ *p* = .014	130 ∙∙ *p* < .001†
MCA proximal	0.29 ∙∙ *p* = .881	- 1.45 ∙∙ *p* = .451	50 ∙∙ *p* = .010†
intermediate	- 1.82 ∙∙ *p* = .379	- 3.90 ∙∙ *p* = .061	100 ∙∙ *p* < .001†
distal	- 3.93 ∙∙ *p* = .068	- 5.69 ∙∙ *p* = .010†	170 ∙∙ *p* < .001†
PCA proximal	- 3.43 ∙∙ *p* = .068	- 4.90 ∙∙ *p* = .010†	100 ∙∙ *p* < .001†
intermediate	- 5.99 ∙∙ *p* = .006†	- 7.64 ∙∙ *p* = .001†	160 ∙∙ *p* < .001†
distal	- 8.52 ∙∙ *p* < .001†	- 9.40 ∙∙ *p* < .001†	250 ∙∙ *p* < .001†
Total gray matter	- 3.15 ∙∙ *p* = .100	- 4.57 ∙∙ *p* = .019	110 ∙∙ *p* < .001†

For each ROI, estimated cross-sectional regression coefficients and *p*-values are shown. †*p*<0.01. ACA, MCA and PCA refer to the flow territories perfused by the anterior, middle and posterior cerebral arteries respectively, corresponding to Fig 2. CBF = cerebral blood flow, M>F stands for higher mL/100g/min in men compared to women, ATT = arterial transit time

### Reliability estimation

**Fig 4:**
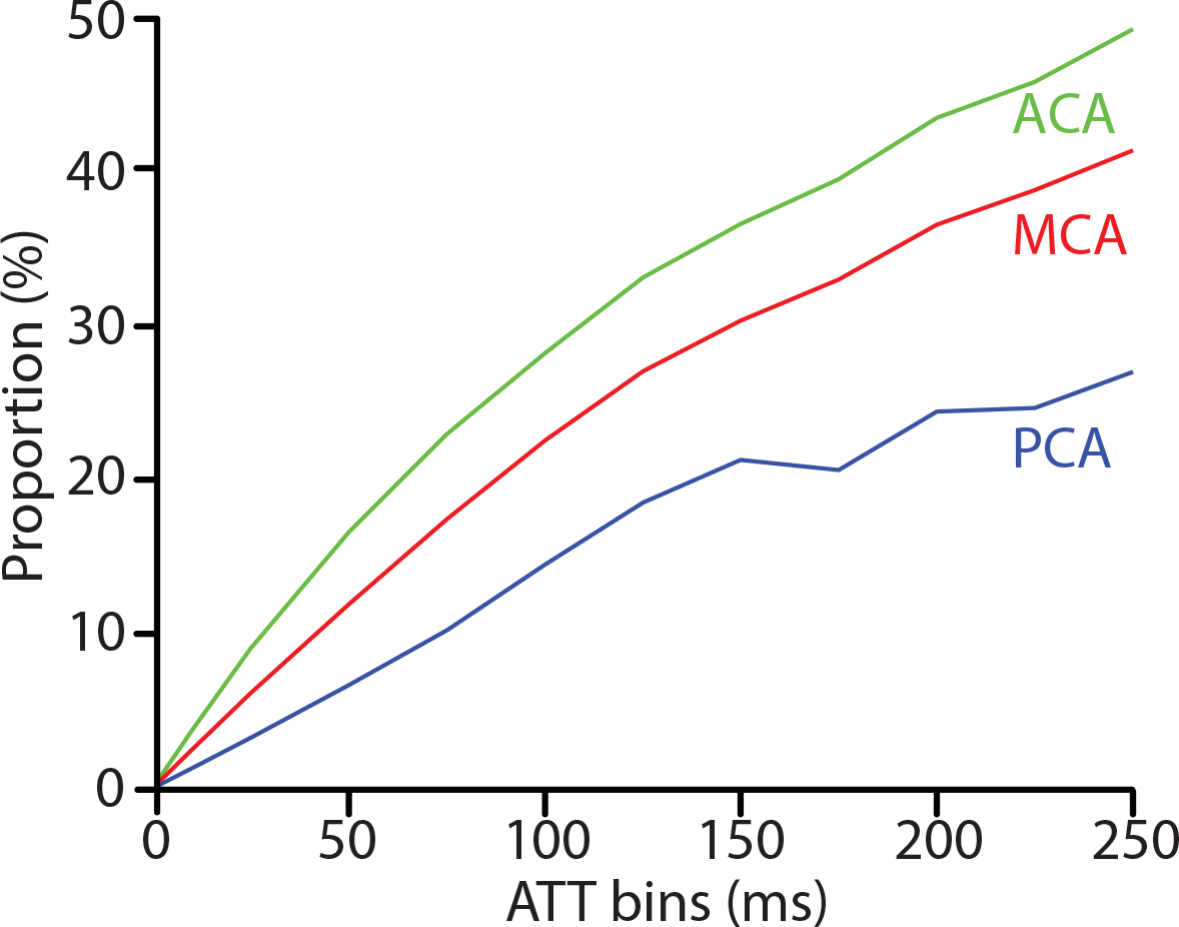
Proportion of arterial transit time (ATT)−values (y-axis) measured by FEAST that is equal to the post-label delay (PLD), for bin sizes 1–250 ms (x-axis), for flow territories perfused by the anterior (ACA), middle (MCA) and posterior cerebral artery (PCA).

To quantify the reliability of FEAST-based ATT values in this population, a post-hoc analysis was performed to search for voxels where the measured ATT lay in the same bin as the effective PLD, for bin sizes ranging from 1–250 ms. With bin sizes of 50–100 ms, the proportions of voxels with artifactual ATT values were 17–28% (ACA), 12–23% (MCA) and 7–14% (PCA) (Fig 4).

## Discussion

The results of this study prove the feasibility of ASL-based CBF_crushed_ and FEAST-based ATT measurements within acceptable scanning time in a population of elderly with hypertension. These perfusion parameters showed stronger correlations with age and gender compared to conventional CBF measurements, demonstrating the potential value of CBF_crushed_ and ATT for group analyses. The observation that the variation of perfusion parameters within flow territories was comparable with the variation of the total gray matter, suggests that the reliability of crushing and FEAST within flow territories is sufficient on a group level, and no further spatial averaging is required. For large clinical imaging studies in elderly with hypertension, therefore, there appears to be potential to study more perfusion parameters than CBF alone.

The ATT maps comply with the trajectory of the cerebral vessels, with shortest ATTs where the vessels enter the cerebrum and longest at the superior-posterior watershed area [23]. The resulting ATT-based flow territories can be used as ROIs in future studies if hypothesized perfusion effects are restricted to certain flow territories only, or if spatial averaging is required when an anatomical structure is too small considering ASL limitations in terms of SNR or spatial smoothing [11]. Potential applications include the investigation of a vascular component to degeneration of the dementia-related regions precuneus and hippocampus, which are supplied by the distal ACA and PCA [24,25]. The fact that distal flow territories demonstrated the largest variation and strongest correlations, could be due to the fact that these regions are most vulnerable to inadequacy of arterial supply due to cerebrovascular pathology [26]. In addition, whole-brain vascular effects across the population can be envisioned to accumulate in the distal flow territories. This suggests that perfusion measurements in distal flow territories have the highest statistical power to detect vascular effects, even if these effects are expected to be distributed across the total brain.

The observed correlations of the perfusion parameters with age lie within the range of previously reported values, whereas the differences with gender were relatively small compared to previous values [27–30]. These correlation differences with previous studies may result from our specific population of high age and hypertension [31,32].

The observation that the population variation of ATT was much smaller than the variation of CBF could originate from the fact that the physiological perfusion fluctuations affect CBF more than ATT. Methodologically, the measured physiological variation can be expected to be similar for ΔM' (CBF_crushed_) and ΔM (CBF_non-crushed_), and will thus not propagate into the FEAST-based ATT measurement, which is derived from the ratio of the two perfusion scans (Fig 1). Likewise, uncertainties in quantification parameters such as blood T1 are expected to have affected the CBF measurements more than the FEAST-based ATT measurements [6]. The smaller extent of random variation of ATT could explain its stronger correlations with age and gender, indicating that ATT measured by FEAST can be a more sensitive parameter for physiological correlations than CBF.

Our study includes the following limitations. The main drawback for the quantification of ATT using the FEAST method, is that the dichotomization of macro‒ and micro‒vascular compartments is based on a single pre-defined velocity cutoff (Fig 1). We have employed a conservative velocity cutoff of 50 mm/s, which is required to retain sufficient SNR in the ASL readout of the CBF_crushed_ measurement (Fig 1) [11]. The drawback of this high velocity cutoff is that CBF_crushed_ is more similar to CBF_non-crushed_, which is illustrated by the fact that the median images of both CBF parameters have a similar visual appearance. Consequentially, the micro‒vascular compartment is defined more proximal, resulting in ATT-values that are more weighted towards the macro-vascular ATT than would be the case with lower velocity cutoffs as implemented in the original FEAST method.

Another drawback of the FEAST method, is that its ATT estimates depend on the selected PLD. The selected PLD ideally should be long enough for labeled blood to arrive in the imaging voxel (i.e. PLD>macro-vascular ATT), but short enough to image before all the labeled blood enters the microvasculature (i.e. PLD<micro-vascular ATT). When the PLD is shorter than the macro-vascular ATT, there will be no labeled signal in either the crushed or non-crushed acquisition, and the division of noise with noise will lead to a Cauchy distribution around a mean ratio of 1, corresponding to an ATT that is equal to the PLD where in reality the ATT is longer. On the other hand, in the case that the PLD is longer than the micro-vascular ATT, all label will already have arrived in the micro-vascular compartment and the signal in both crushed and non-crushed acquisitions will be similar. This again leads to a ratio of 1, corresponding to an ATT equal to the PLD where in reality the ATT is shorter. Both situations would lead to narrow ATT histograms with minimum values coinciding with the PLD.

To which extent the skewness in the ATT histograms reflects the physiological distribution of ATT within flow territories, or is due to too long macro-vascular ATT or too short micro-vascular ATT relative to the PLD, cannot be differentiated with these data. According to our comparison of the FEAST-based ATT values with PLD-values (Fig 4), we estimate that our values are artifactual in maximally 10–25% of the voxels. The fact that the amount of artifactual voxels was highest for the ACA and lowest for the PCA ‒ acknowledging the generally longer ATT in the PCA [4,10] ‒ suggests that FEAST measurement errors in this study mainly stem from the PLD being longer than the micro-vascular ATT rather than the PLD being shorter than the macro-vascular ATT. Apparently, our PLD was sufficiently long for the macro-vascular ATT in this population [10]. This is in agreement with the observations in the original FEAST papers [6,10], where good results were obtained in healthy volunteers using FEAST with a PLD range as low as 300–700 ms [6]. In a 42-year old subject with left MCA occlusion [10] and a 82-year old patient with left ICA and left MCA stenosis [6], FEAST previously provided satisfactory results with PLD ranges of 1000–1300 ms and 800–1200 ms respectively.

Nevertheless, it is remarkable that the PCA exhibited the lowest proportion of artifactual ATT-values (Fig 4) but also the largest CV (Fig 3F). This large ATT variation could be explained by the relatively large anatomical variability of the posterior circulation. Up to 36% of the population has a fetal-type posterior circle of Willis, in which the PCA is partly or fully perfused by the carotid instead of the basilar artery [33], bearing shorter and more heterogeneous ATT [34]. This physiological ATT variability can be expected to have enlarged the between-subject ATT variation, but does not necessarily lead to artifactual ATT-values.

To satisfy PLD criteria for both CBF and ATT measurements, we applied a PLD that is a trade-off between conventional CBF measurements and FEAST-based ATT measurements. Therefore, our mean PLD (1800 ms) and labeling duration (1650 ms) were shorter than advised for CBF measurements in this population (2000 ms and 1800 ms respectively) in the recent ASL consensus paper [11]. It can be expected that the fact that we observed stronger correlations for CBF_crushed_ compared to CBF_non-crushed_ is, at least partly, due to our shorter PLD [6,10].

It should be noted that because of the abovementioned velocity cutoff and PLD-limitations, the ATT-values presented in this study should not be referred to as absolute values. Because of logistic reasons and time constraints we were unable to perform multi-TI measurements in a small subset of subjects, which would have allowed us to calibrate the FEAST-based ATT-values [6,10]. Nevertheless, our main results ‒ the spatial distribution of ATT and the correlations with age and gender ‒ can be expected to remain largely the same when such a calibration would have been carried out [6].

Partial volume effects form another potential limitation, considering the relatively large voxel size of 3.75x3.75x7 = 98.4 mm^3^. However, acquisition-related partial volume effects are expected to be equal for CBF_crushed_, CBF_non-crushed_ and ATT maps, as these are all acquired with the same resolution and orientation. Hence, comparisons between these acquisitions are not expected to be significantly influenced by partial volume effects. In the current study, the labeling plane was fixed 8.3 cm inferior to the AC-PC line. Although this location has been shown to provide optimal labeling efficiency in young healthy volunteers [13], the increased curvature of extracranial vessels in the elderly may have decreased the mean labeling efficiency and increased the variability of the labeling efficiency [11].

In conclusion, we have shown the feasibility and value of combined measurements of CBF and FEAST-based ATT for large imaging studies in elderly with hypertension. The obtained flow territories can be used in future studies to identify regional vascular effects. On a group level, crushing can improve correlations with physiological parameters such as age and gender. The high physiological correlations of ATT suggest that this perfusion parameter can be more relevant than conventional CBF measurements. However, the PLD should be carefully selected and one should account for the possible under- and overestimation of ATT. These data encourage future clinical imaging studies in elderly with hypertension to investigate multiple MRI perfusion parameters, instead of focusing at CBF only.
